# CTSK and PLAU as Prognostic Biomarker and Related to Immune Infiltration in Pancreatic Cancer: Evidence from Bioinformatics Analysis and qPCR

**DOI:** 10.1155/2023/3914687

**Published:** 2023-11-29

**Authors:** Yuntao Ding, Zhangzuo Li, Huizhi Wang, Qi Wang, Han Jiang, Zhengyue Yu, Min Xu

**Affiliations:** ^1^Department of Gastroenterology, Affiliated Hospital of Jiangsu University, Jiangsu University, Zhenjiang, China; ^2^Department of Cell Biology, School of Medicine, Jiangsu University, Zhenjiang, China

## Abstract

Pancreatic adenocarcinoma (PAAD) is a malignancy with the highest mortality rate worldwide. There is a pressing need for novel biomarkers that can facilitate early detection and serve as targets for therapeutic interventions beyond the commonly utilized CA199 marker. This study utilized microarray datasets (GSE15471, GSE62165, and GSE28735) from the Gene Expression Omnibus (GEO) to identify differentially expressed genes (DEGs) and construct a protein-protein interaction network using STRING and Cytoscape. Hub genes were selected using BiNGO. Expression profiles and clinical data from the Cancer Genome Atlas (TCGA) were then used to compare the expression levels of CTSK and PLAU in pancreatic cancer and healthy pancreatic tissues via the Wilcoxon rank-sum test, with further validation using qPCR. Functional enrichment analysis was conducted to explore potential signaling pathways and biological functions. Prognostic values were assessed by the Kaplan-Meier and Cox regression analyses, and an overall survival (OS) nomogram was created to predict 1-, 2-, and 3-year survival after cancer diagnosis. The infiltration of immune cells was evaluated by single-sample gene set enrichment analysis. The methylation status of both genes was analyzed using the UALCAN and MethSurv databases. The results demonstrated that CTSK and PLAU were overexpressed in pancreatic cancer and that the hypomethylation status of both genes was associated with a poor prognosis. The overexpression of both genes was positively correlated with various immune cells, and functional enrichment analysis revealed that they were associated with immune cell infiltration. Besides, the effects of PLAU on the migration and invasion of pancreatic cancer cells were also verified by scratch and transwell experiments. Consequently, CTSK and PLAU have potential as prognostic biomarkers for pancreatic cancer.

## 1. Introduction

Pancreatic adenocarcinoma (PAAD) is an increasingly prevalent malignancy and a leading cause of cancer-related mortality, exhibiting a low 5-year survival rate of approximately 10% and a high mortality rate [1]. The incidence and mortality of PAAD are also increasing annually in comparison to some developed countries [2]. CA19-9 is a well-established circulating biomarker for pancreatic cancer [3], but its sensitivity as a diagnostic biomarker is limited to approximately 80% [4]. Consequently, there is a need for new biomarkers that can facilitate early detection. Differential gene expression analysis, carried out using bioinformatic and microarray techniques, has identified numerous differentially expressed genes (DEGs). However, discerning credible results from independent microarray analyses' false-positive rates can be challenging. As such, we utilized the Gene Expression Omnibus to choose and download three microarray datasets (GEO) comparing pancreatic cancer tissues and noncancerous tissues to acquire DEGs.

Notably, KRAS and TP53 mutations are two significant drivers of pancreatic cancer and play essential roles in tumor invasion and transformation [5]. KRAS, which can be regarded as a rheostat in cells due to its dependence on the number of active molecules [6], can be utilized as a biomarker to predict the prognosis of PAAD or as a target for tumor therapy [7]. TP53, on the other hand, is a tumor suppressor whose mutations suppress DNA repair-stimulatory and apoptosis-inducing functions, leading to the development of PAAD [8]. Its mutation enhances the cancer's activities by up to 70% [5]. ARF6-AMAP1 pathways, empowered by KRAS/TP53 mutations, may foster fibrosis in PAAD and lead to immune evasion [9].

So, while the molecular biology of KRAS and TP53 has been sufficiently explored, other genes are also critical to PAAD. Subsequently, using TCGA database, we performed Gene Ontology (GO), GSEA from the Kyoto Encyclopedia, and protein-protein interaction network (PPI) to gain a better understanding of the molecular mechanism of PADD development. We employed PCR to demonstrate the differential expression of both genes in the PAAD cell line, and we studied the immune infiltration and methylation-related indicators of the target genes. This study is aimed at employing bioinformatics to investigate and comprehend the expression of PLAU and CTSK in pancreatic cancer, as well as their relationship with clinicopathological and prognostic significance, underlying molecular mechanisms, and immune cell infiltration, to aid physicians.

To summarize, our findings suggest that CTSK and PLAU may serve as novel potential biomarkers for pancreatic cancer. The materials and methods used are detailed below.

## 2. Methods and Materials

### 2.1. Data Collection and Processing

We obtained three datasets of gene expression: GSE15471, GSE62165, and GSE28735 from the Gene Expression Omnibus (GEO) database, which were generated using the Affymetrix Human Genome U133 Plus 2.0 Array. We converted the probe data to the corresponding gene symbols using the annotation information on the platform. The GSE15471 dataset comprised of 39 pairs of pancreatic adenocarcinoma (PAAD) tumors and normal pancreatic tissues. The GSE62165 dataset included 118 PDAC samples and 13 control samples, while the GSE28735 dataset contained 45 PDAC tissues and 45 adjacent nontumorous counterparts. Additionally, we downloaded 33 kinds of tumor project STAR process RNAseq data from TCGA database (https://portal.gdc.cancer.gov). The relevant data on normal tissues and cells were downloaded from the Genotype-Tissue Expression (GTEx) database. Transcripts per million reads (TPM) are used to standardize the HTSeq FPKM Level 3 data. R software v4.2.1 was used for statistical analysis. Wilcoxon's rank-sum test was used to detect the data of the two groups (ns, *p* ≥ 0.05; ^∗^*p* < 0.05; ^∗^*p* < 0.01; ^∗^*p* < 0.001).

### 2.2. Differentially Expressed Gene

We utilized the GEO2R tool to determine the differentially expressed genes (DEGs) between PAAD tissues and matched normal pancreatic tissue samples. GEO2R is an interactive web-based tool that allows for the comparison of two or more sets of samples from the GEO series to identify genes that exhibit significant expression changes in different experimental conditions (https://www.ncbi.nlm.nih.gov/geo/geo2r/). To minimize false positives and identify statistically significant genes, we used a combination of *p* values and the Benjamini and Hochberg false discovery rate. We excluded probe sets with mismatched gene symbols or genes with multiple probe sets. logFC (fold change) >1 or <-1 and *p* value < 0.05 were considered statistically significant.

### 2.3. PPI Network Construction and Module Analysis

We employed an online database and interactive gene search tool, STRING (http://string-db.org) (version 11.5) [10], to explore the molecular interactions among DEGs. We selected interactions with a composite score of >0.4, and the resulting molecular interaction networks were visualized using Cytoscape (version 3.9.1) [11]. To identify the most significant modules in the protein-protein interaction (PPI) network, we used the Molecular Complex Detection (MCODE) algorithm [12]. We employed the following selection criteria: MCODE scores > 5, degree cut − off = 2, node score cut − off = 0.2, max depth = 100, and *k*-score = 2.

### 2.4. Hub Genes Selection and Analysis

Genes with a rank ≥ 10 were considered hub genes. Coexpressed genes were analyzed through the cBioPortal online platform (http://www.cbioportal.org) [12, 13]. To identify the biological processes associated with the hub genes, we used the Biology Network Gene Ontology tool (BiNGO) plugin (version 3.0.5) for Cytoscape [14]. The hierarchical clustering of hub genes was constructed using the UCSC Cancer Genomics Browser (http://genome-cancer.ucsc.edu) [15]. The overall and disease-free survival of hub genes were analyzed using the Kaplan-Meier curves on cBioPortal. Furthermore, we assessed the correlation between the expression of PLAU and CTSK and their seed genes in PAAD with Spearman's correlation analysis.

### 2.5. Functional Enrichment Analysis

We utilized the R package clusterProfiler (4.4.4) and GOplot (1.0.2) to conduct Gene Ontology (GO), Kyoto Encyclopedia of Genes and Genomes analyses (KEGG), and gene set enrichment analysis (GSEA) of the differentially expressed genes (DEGs). We considered *p* values < 0.05 and false detection rates (FDR) <0.25 to identify statistically significant functions or signal pathways (Subramanian et al., 2005; Yu et al., 2012).

### 2.6. Diagnostic Value Analysis

The receiver operating characteristic (ROC) and time-dependent receiver operating characteristic curve were used to evaluate the diagnostic value of PLAU and CTSK in PAAD. The pROC package (version 1.18.0) was used to analyze the data, and the results were visualized by ggplot2 (version 3.3.6). By default, the pROC package corrects the outcome order of the data, and *p* < 0.05 is considered statistically significant.

### 2.7. Clinical Significance of PLAU and CTSK in PAAD

Calibration, nomogram, and forest map were used for further clinical significance analysis of PLAU and CTSK in PAAD. The survival package (version 3.3.1) was used for proportional hazard hypothesis testing and Cox regression analysis, and the rms package (version 6.3-0) was used for calibration analysis and visualization. The survival package was used for proportional hazard hypothesis testing and Cox regression analysis, and the rms package was used to construct and visualize the nomogram correlation model. Forest map visualization was performed using ggplot2 (version 3.3.6).

### 2.8. DNA Methylation Analysis

To explore the potential mechanisms of CTSK and PLAU in pancreatic cancer, we assessed the methylation status of their promoters using the UALCAN database (Chandrashekar et al., 2017). Furthermore, we used the MethSurv database, an online tool for multivariate survival analysis based on DNA methylation data, to evaluate the predictive value of the methylation levels of PLAU and CTSK (Modhukur et al., 2018).

### 2.9. Immune Infiltration Analysis

The abundance of 24 immune cells was quantified to evaluate the level of immune infiltration in pancreatic cancer. The GSEA algorithm was applied to identify the relative enrichment of immune cells using a single probe utilizing the GSV R package (Bindea et al., 2013). Spearman's correlation analysis was used to examine the association between the expression levels of PLAU and CTSK and immune cells, while the Wilcoxon rank-sum test was used to compare the differences in the level of immune infiltration between the high and low expression groups of PLAU and CTSK.

### 2.10. Cell Line and Cell Culture

Pancreatic cancer cell lines BxPc-3, PaTu8988, PANC-1, and mia-Paca2 and normal pancreatic cell line HPDE6-C7 were stored by the Central Laboratory of the Affiliated Hospital of Jiangsu University and were kindly provided by Second Military Medical University in Shanghai. All cell lines were cultured in DMEM (Hyclone, Beijing, China) supplemented with 10% fetal bovine serum (Gibco, Carlsbad, CA, USA) at 37°C in a humidified incubator with a 5% CO_2_ supply.

### 2.11. RNA Extraction and Real-Time PCR

Trizo Plus (Takara, Shiga, Japan) was used to extract the total RNA. According to the manufacturer's specification, reverse transcription was performed using the RevertAid First Strand cDNA Synthesis Kit (Thermo, Waltham, MA, USA). Real-time PCR was performed in triplicate in 20 ml reactions with iQ SYBR Premix Ex Taq Perfect Real Time (Bio-Rad Laboratories, Inc., Hercules, CA, USA), 50 ng of first-strand cDNA, and 0.4 mg of each primer. Actin was selected as a housekeeping gene. The primer pairs used for the amplification of the CTSK, PLAU, and human action were as follows: CTSK forward primer: 5′- GTGTCTGAGAATGATGGCTGTGGAG-3′, and reserve primer: 5′- TGCCTTGCCTGTTGGGTTGTAC-3′, and PLAU: forward primer:5′- TCGCTCAAGGCTTAACTCCAACAC-3′, and reserve primer: 5′- ACGGATCTTCAGCAAGGCAATGTC-3′, actin forward primer:5′- CACGAAACTACCTTCAACTCC-3′, and reserve primer: 5′- CATACTCCTGCTTGCTGATC-3′. Samples were cycled once at 95°C for 2 min and then subjected to 35 cycles of 95°C, 56°C, and 72°C for 30 s each. The relative mRNA content was calculated using the 2 − ΔΔCT method with GAPDH as an endogenous control.

### 2.12. Cell Migration and Invasion Assay

A scratch test was used to detect cell migration ability according to the reagent manufacturer's protocol. Transwell assays were performed using transwell inserts (Corning, Corning, New York, USA) containing 8 m permeable wells according to the manufacturer's protocol. Transfected PaTu8988 and BxPc-3 cells were harvested, resuspended in serum-free medium, and transferred to 8 m permeable wells (B100 000 cells per well). The cells were then incubated with a culture medium containing 10% FBS for 24 h before detection. The cells on the upper surface were scraped off, and the migrating cells on the lower surface were fixed and stained with 0.05% crystal violet for 30 min. Finally, five independent fields per transwell were counted, and the average number of cells per field is represented in the figure. To assess cell invasion, 100,000 cells were seeded in Matrigel-coated transwell inserts (BD Bioscience, Corning, NY, USA) in a serum-free medium. Cells were then treated similarly to cell migration assays.

### 2.13. Statistical Analysis

All statistical analyses were performed using R (version 3.6.3). Wilcoxon's rank-sum test and paired sample *t*-test were used to assess the statistical significance of PALU and CTSk expression in unpaired and paired tissues. Associations between clinical features and their expression were assessed using the Wilcoxon rank-sum test and logistic regression. All tests were two-tailed, and *p* values < 0.05 were considered statistically significant.

## 3. Results

### 3.1. Identification of DEGs in PAAD

**Figure 1:**
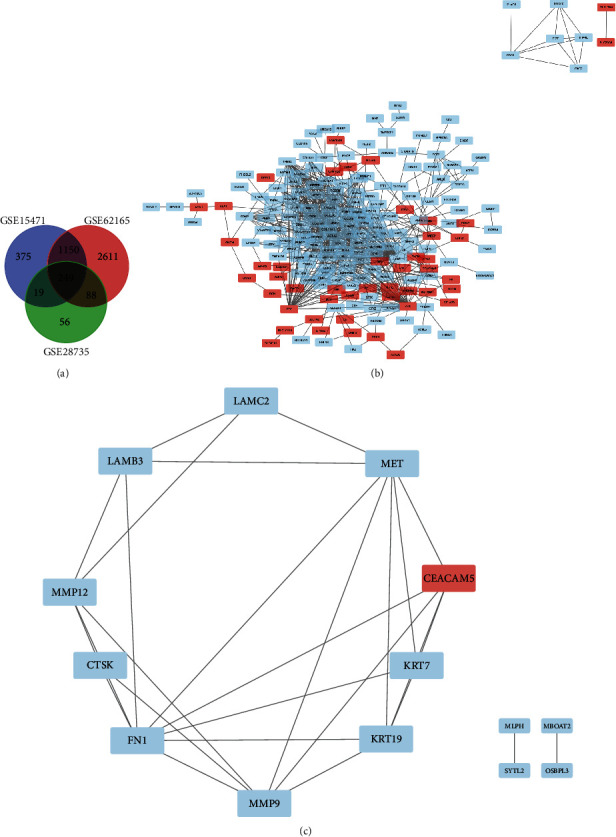
Venn diagram, PPI network, and the most significant module of DEGs. (a) Three microarray databases GSE15471, GSE62165, and GSE28735 were selected from GEO with logFC (fold change) >1 or <-1 and adj. *p* value < 0.05. The 3 datasets showed an overlap of 249 genes. (b) The PPI network of DEGs was constructed using Cytoscape. (c) We select CTSk as the most significant module (7 nodes and 14 edges) and draw the PPI network. Upregulated genes are marked in light red; downregulated genes are marked in light blue.

After analysing the microarray results, DEGs (375 in GSE15471, 2611 in GSE62165, and 56 in GSE28735) were identified. Among the 3 datasets were 249 genes shown in the Venn diagram (Figure 1(a)), including 183 downregulated genes and 66 upregulated genes among pancreatic ductal adenocarcinoma tumors that matched standard pancreatic tissue samples.

### 3.2. PPI Network Construction and Module Analysis

Using Cytoscape, we drew the PPI network of DEGs and identified the most important module (Figures 1(a) and 1(c)). KEGG and GO's functional analyses of genes involved in this module were analyzed using the R package cluster profile. Results showed that genes in this module were mainly enriched in an extracellular matrix organisation, laminin complex collagen binding, and the PI3K-Akt signaling pathway (Table 1).

### 3.3. KEGG and GO Enrichment Analyses of DEGs

**Figure 2:**
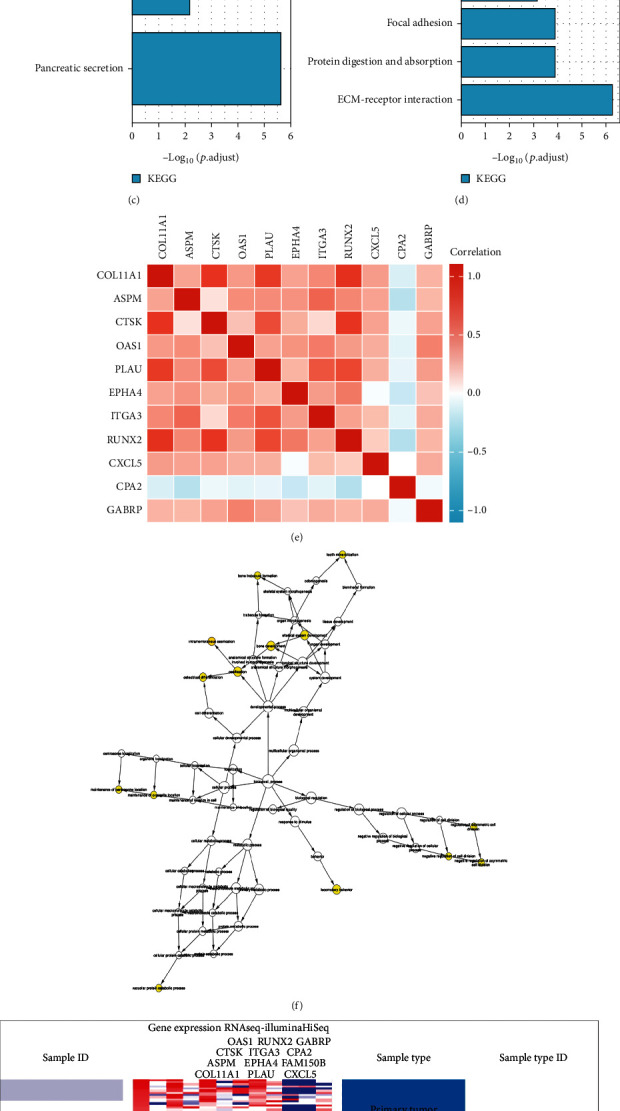
Analysis of DEGs and hub genes. (a, b) GO enrichment of all upregulated and downregulated DEGs. (c, d) KEGG enrichment between all upregulated and downregulated DEGs. (e) We print a correlation heat map of hub genes with degree ≥ 10. (f) Analysis of the biological process of constructing pivotal genes by BiNGO. The node color depth is the *p* value after ontology correction. Node size refers to the number of genes involved in the ontology. *p* < 0.05 was considered statistically significant. (g) Hierarchical clustering of hub genes with degree ≥10 was drawn by UCSC. From left to right were sample ID, gene expression, sample type, and sample type ID. The upregulation of genes is marked in red; the downregulation of genes is marked in blue.

To analyze the biological classification of DEGs, GO analysis showed that for all upregulated DEGs, they were mainly enriched in toxic substance binding, exopeptidase activity, serine-type endopeptidase activity, serine hydrolase activity, serine-type peptidase activity in cell component (CC), asymmetric synapse, platelet alpha granule, postsynaptic density, platelet alpha granule lumen, and neuron-to-neuron synapse in molecular function (MF). For downregulated DEGs, they were mainly enriched in metalloendopeptidase activity, collagen binding, integrin binding, and extracellular matrix structural constituent in molecular function (MF). In the cell component (CC), they are mainly enriched in the basement membrane, endoplasmic reticulum lumen, extracellular matrix component, and the collagen-containing extracellular. And in biological processes (BP), they are mainly enriched in the formation of the primary germ layer, collagen fibril organisation, extracellular structure organization, and extracellular matrix organization. KEGG pathway analysis revealed that the downregulated DEGs were mainly enriched in protein digestion and absorption and pancreatic secretion. In contrast, the majority of the PI3K-Akt signaling pathway, amoebiasis, focal adhesion, protein digestion and absorption, and ECM-receptor interaction were concentrated in the elevated DEGs (Figures 2(a)–2(d)).

### 3.4. Hub Gene Selection and Analysis

**Figure 3:**
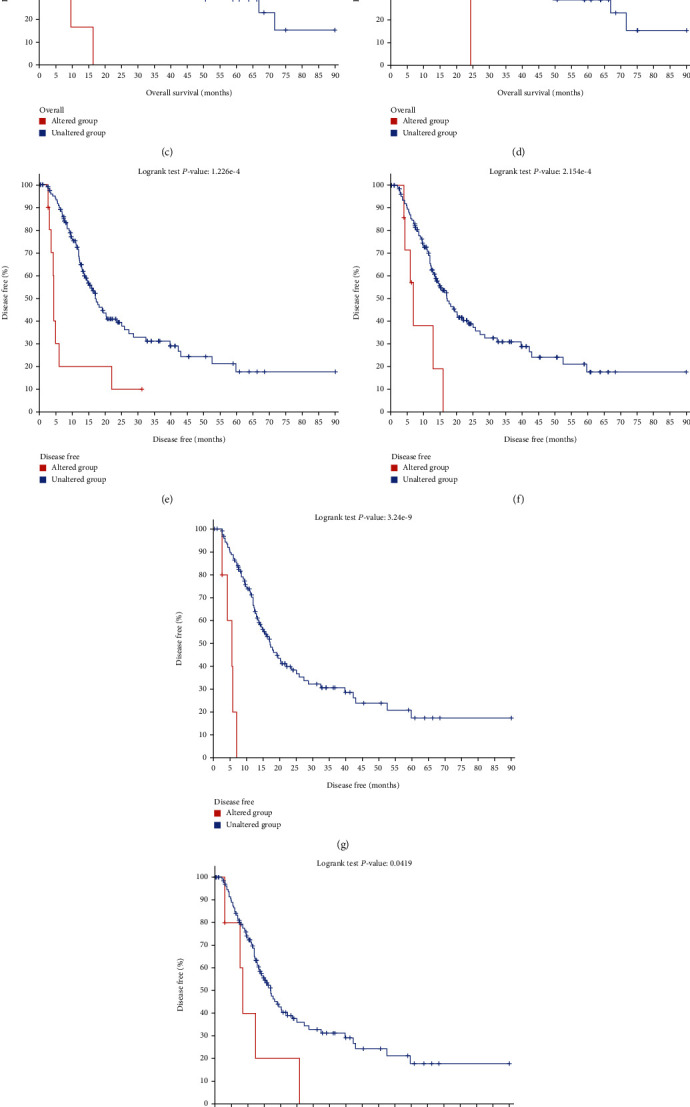
Overall survival and disease-free survival analyses of hub genes by cBioPortal online platform. *p* < 0.05 was considered statistically significant. (a–d) Overall survival for ASPM, OAS1, PLAU, and CTSK. (e–i) Disease-free survival for ASPM, OAS1, PLAU, TGA3, and CTSK.

We select 12 genes with degrees ≥ 10 as hub genes. In Table 2, we make a note of their names and functions. According to TCGA database, the correlation heat map was made by the supermen correlation coefficient, showing that those hub genes were correlated (Figure 2(e)). The biological process analysis of the hub genes is shown in Figure 2(f). We analyze the biological process of the hub genes. Hub genes could be clearly distinguished by hierarchical clustering between pancreatic ductal adenocarcinoma tumors and matching normal pancreatic tissue samples (Figure 2(g)). The cBioPortal online database was used explore overall survival and disease-free survival by using the Kaplan-Meier curve. Results showed that the expression of ASPM, OAS1, PLAU, and CTSK may lead to a poor prognosis for overall survival (Figures 3(a)–3(d)). We can also find that the expression of ASPM, OAS1, PLAU, and ITGA3 may lead to a poor prognosis for disease-free survival (Figures 3(e)–3(i)). Among these genes, COL1A1, PLAU, and CTSK showed the highest node genes, which may play important roles in the carcinogenesis or progression of PAAD. As COL1A1 has been well studied in pancreatic cancer, we selected PLAU and CTSK, which have shown reductions in overall and disease-free survival. The hub gene PLAU whose overall and disease-free survival is statistically significant (*p* = 3.24*e* − 9 for disease-free survival and *p* = 3.146*e* − 5 for overall survival) (Figures 3(c) and 3(g)); however, the hub gene CTSK whose overall and disease-free survival is not statistically significant (*p* = 0.716 for disease-free survival and *p* = 0.804) for overall survival. (Figures 3(d) and 3(i)). Further confirmation of CTSK clinical significance in overall survival and disease-free survival by obtaining patient samples is needed in follow-up studies.

### 3.5. Elevated Expression of CTSK and PLAU in PAAD for Bioinformatics Analysis

**Figure 4:**
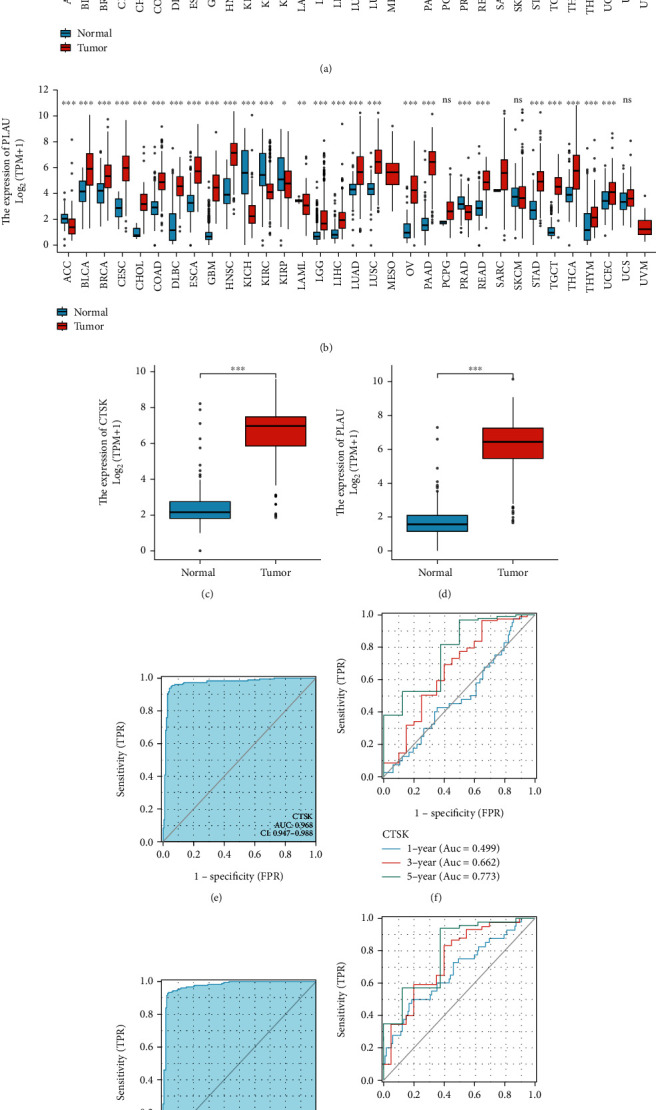
Clinical relevance of CTSK and PLAU. (a, b) Pancancer analysis of PLAU and CTSK. (c, d) Expression of PLAU and CTSK in PAAD. (e, f) ROC curve of PLAU and CTSK. (g, h) Time-dependent ROC curve.

Pancancer analysis suggested that PLAU and CTSK were differentially expressed in pancreatic cancer and other cancers such as kidney chromophobe, stomach adenocarcinoma, and v = ovarian serous cystadenocarcinoma (Figures 4(a) and 4(b)). The expression of PLAU and CTSK was significantly higher in pancreatic cancer samples than in normal breast tissues (*p* < 0.001) (Figures 4(c) and 4(d)).

### 3.6. Diagnostic Value Analysis

In addition, the ROC curve showed that PLAU and CTSK had excellent predictive ability to distinguish pancreatic cancer, with an area under the curve of 0.968 (95% CI = 0.947-0.988) for CTSK and an area under the curve of 0.976 (95% CI = 0.961-0.991) for PLAU (Figures 4(e) and 4(g)). Additionally, we created time-dependent ROC curves to assess further and compare the predictive performance of PLAU and CTSK, with the area under the ROC curve (AUROC) as the criterion. Results demonstrated that CTSK and PLAU both had excellent prediction for PAAD, with an area under the curve of 0.499, 0.662, and 0.773 in 1, 3, and 5 years for CTSK and an area under the curve of 0.672, 0.744, and 0.792 in 1, 3, and 5 years for PLAU (Figures 4(f) and 4(h)).

### 3.7. Prognostic Value of CTSK and PLAU in PAAD

To find prognostic factors, univariate and multivariate Cox regression analyses were performed.

**Figure 5:**
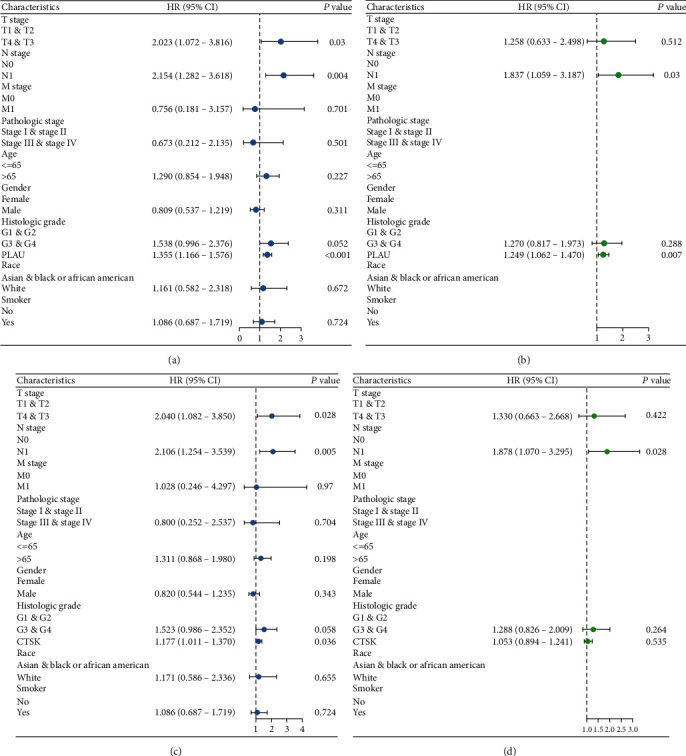
Prognostic values of PLAU and CTSK expression in patients with PAAD by univariate analysis and multivariate analysis. (a, b) Prognostic values of PLAU expression by univariate analysis and multivariate analysis. (c, d) Prognostic values of CTSK expression by univariate analysis and multivariate analysis.

The results of the univariate analysis demonstrated that for PLAU, T4&T3 stage (adjusted HR = 2.023, 95% CI = 1.072–3.816, *p* = 0.03), N1 (adjusted HR = 2.154, 95% CI = 1.282–3.618, *p* = 0.004), and the expression of PLAU (adjusted HR = 1.355, 95% CI = 1.166–1.576, *p* < 0.01) were independent factors of OS in patients with PAAD, while for CTSK, T4&T3 stage (adjusted HR = 2.0403, 95% CI = 1.082–3.850, *p* = 0.028), N1 (adjusted HR = 2.106, 95% CI = 1.254–3.539, *p* = 0.005), and the expression of CTSK (adjusted HR = 1.177, 95% CI = 0.586–2.326, *p* = 0.036) were independent factors of OS in patients with PAAD (Figures 5(a) and 5(b)). While the results of the multivariate analysis demonstrated that for PLAU, N1 (adjusted HR = 1.837, 95% CI = 1.059–3.187, *p* = 0.03) and the expression of PLAU (adjusted HR = 1.249, 95% CI = 1.062–1.470, *p* = 0.007) were multivariate factors of OS in patients with PAAD, while for CTSK, N1 (adjusted HR = 1.878, 95% CI = 1.070-3.295, *p* = 0.028) was independent factors of OS in patients with PAAD (Figures 5(c) and 5(d)).

### 3.8. Construction and Validation of the Nomogram

**Figure 6:**
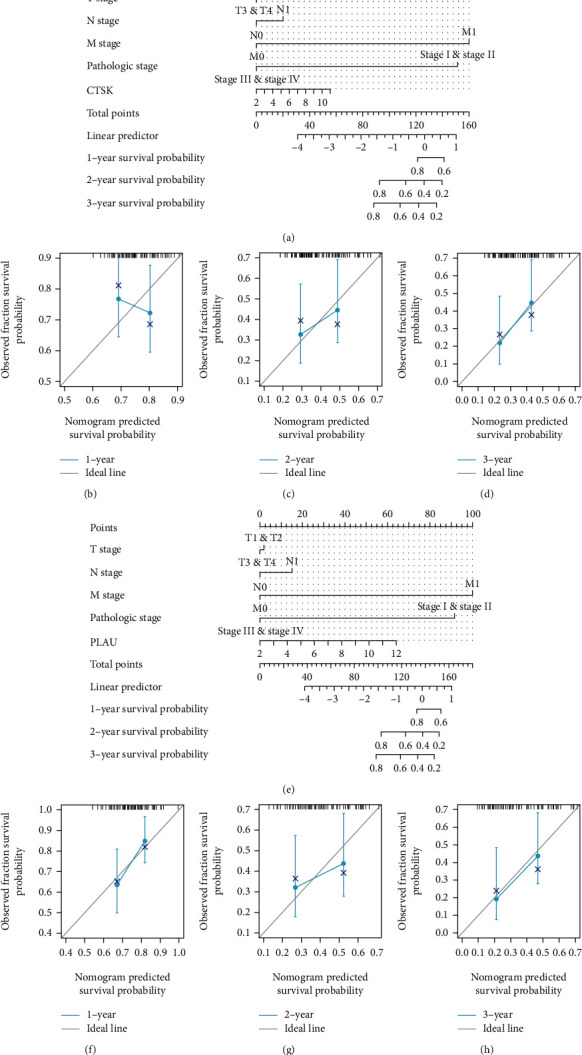
A nomogram and calibration curves for prediction of one-, two-, and three-year overall survival rates of patients with PAAD. (a, e) A nomogram for prediction of one-, two-, and three-overall survival rates of patients with PAAD with high expression of CTSK and PLAU. (b–d, f–h) Calibration curves of the nomogram prediction of one-, two-, and three-year overall survival rates of patients with PAAD with high expression of CTSK and PLAU.

We constructed a nomenclature map based on independent OS factors to predict the (b–d, f–h) prognosis of pancreatic cancer patients. The higher the average score, the worse the prognosis (Figures 6(a) and 6(e)). Furthermore, the predictive performance of the nomogram was assessed using standard curves (Figures 6(b)–6(d) and 6(f)–6(h)). These results suggest that the nomogram is suitable.

### 3.9. Analysis of Related Differentially Expressed Genes of PLAU and CTSK in PAAD

**Figure 7:**
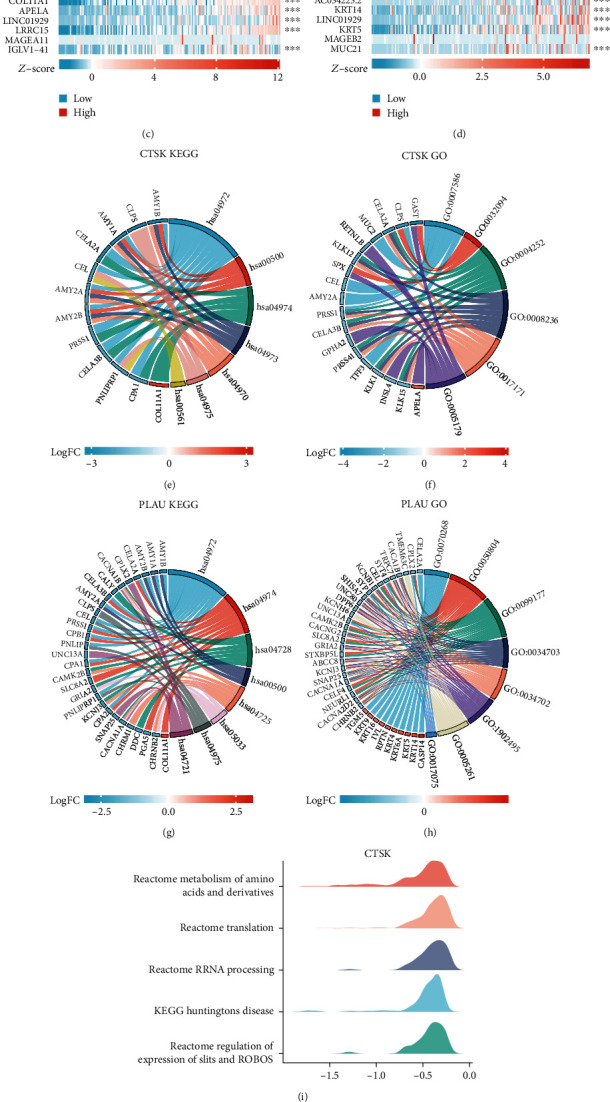
Related differentially expressed genes of PLAU and CTSK. (a–d) The volcano plot and top ten related differentially expressed genes of CTSK and PLAU from TCGA database. (e–j) GO analysis, KEGG analysis, and GSEA of CTSK and PLAU and their coexpression genes.

To further explore the functional enrichment of the two target molecules in pancreatic cancer, the differentially expressed genes (DEGs) of PLAU and CTSK in PAAD were analyzed by the Dseq2 R package. The results showed 125 differentially expressed genes between the high-expression and low-expression CTSK groups, including 20 upregulated genes and 105 downregulated genes (*p* value < 0.05, |Log2 − FC| > 2). Moreover, in PLAU, there were 339 gene expression differences between the high- and low-expression CTSK groups, of which 73 were upregulated and 266 were downregulated (*p* value < 0.05, |Log2 − FC| > 2) (Figures 7(a) and 7(b)). And then, the relationship between the top 5 high and top 5 low DEGs in CTSK (including EPYC, MAGEA4, CASP14, MAB21L2, COL11A1, APELA, LINC01929, LRRC15, MAGEA11, and IGLV1−41) were presented in Figure 7(c), and the relationship between the top 5 high and top 5 low DEGs (including CASP14, MIR205HG, CGB5, S100A2, AC034223.2, KRT14, LINC01929, KRT5, MAGEB2, and MUC21) in PLAU were presented in Figure 7(d).

### 3.10. Function Enrichment for DEGs of CTSK and PLAU in PAAD

For GO enrichment analysis, DEGs of CTSK were mainly enriched in digestion, response to food, serine-type endopeptidase activity, serine-type peptidase activity, serine hydrolase activity, and hormone activity (Figure 7(f)), while PLAU is mainly enriched in cornification, modulation of chemical synaptic transmission, regulation of trans-synaptic signaling, cation channel complex, ion channel complex, transmembrane transporter complex, cation channel activity, and syntaxin-1 binding (Figure 7(h)). For KEGG analysis, CTSK is mainly enriched in pancreatic secretion, starch and sucrose metabolism, protein digestion and absorption, carbohydrate digestion and absorption, salivary secretion, fat digestion and absorption, and glycerolipid metabolism (Figure 7(e)), while PLAU is mainly enriched in pancreatic secretion, protein digestion and absorption, dopaminergic synapse, starch and sucrose metabolism, cholinergic synapse, nicotine addiction, fat digestion and absorption, and synaptic vesicle cycle (Figure 7(g)). In Figures 7(i) and 7(h), we analyzed all DEGs for their GSEA which showed that PLAU and CTSK are connected with Reactome GPCR ligand binding, Reactome G alpha-1 signaling events, Reactome neuronal system, Reactome metabolism of amino acids and derivatives, Reactome class A 1 rhodopsin-like receptors, Reactome metabolism of amino acids and derivatives, Reactome translation, Reactome rRNA processing, KEGG Huntington's disease, and Reactome regulation of expression of Slits and Robos in PAAD. These results suggest that there may be a relationship between these two genes and immune infiltration.

### 3.11. Correlation between Methylation and Expression of PLAU and CTSK

**Figure 8:**
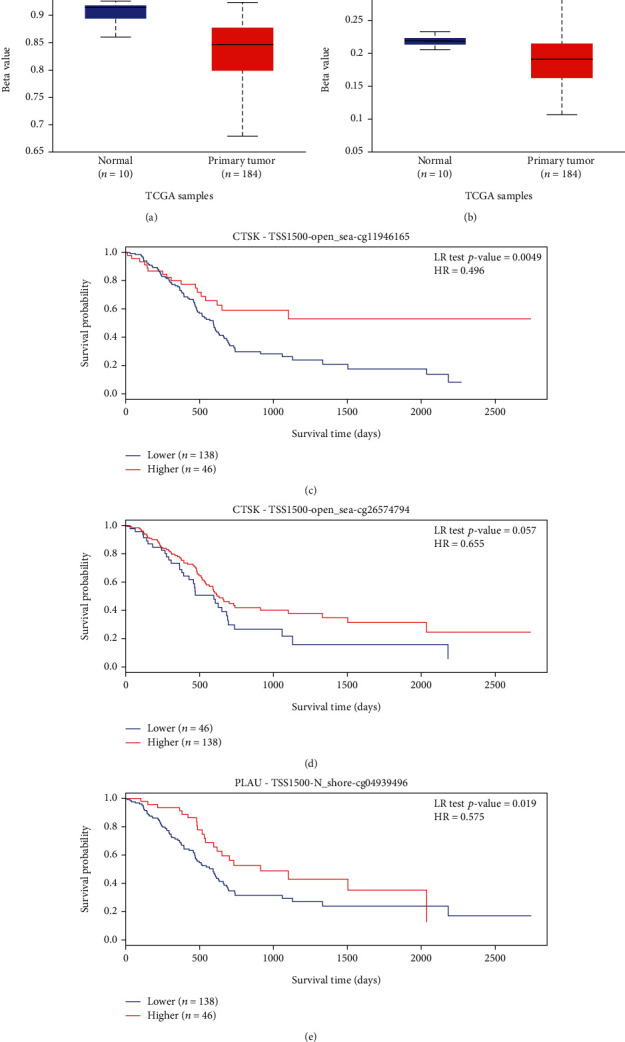
DNA methylation level of CTSK and PLAU and their effect on prognosis of patients with PAAD. (a, b) The promoter methylation level of CTSK and PLAU in PAAD was obtained from the UALCAN database. (c–e) Kaplan-Meier survival curves for several methylation sites of CTSK and PLAU with *p* value < 0.05.

To further characterise the mechanism of PLAU and CTSK overexpression in PAAD, we examined the correlation between PLAU and CTSK expression levels and methylation status. Figures 8(a) and 8(b) clearly show that the DNA methylation of CTSK and PLAU at the promoters is significantly lower than that of normal pancreatic tissue from the UALCAN database. In addition, several methylated sites were associated with poor prognosis in CTSK, including cg1946165 and cg26574794 (Figures 8(c) and 8(d)), while for PLAU, it was cg04939496 (Figure 8(e)).

### 3.12. Correlation between PALU and CTSK Expression and Immune Infiltration

**Figure 9:**
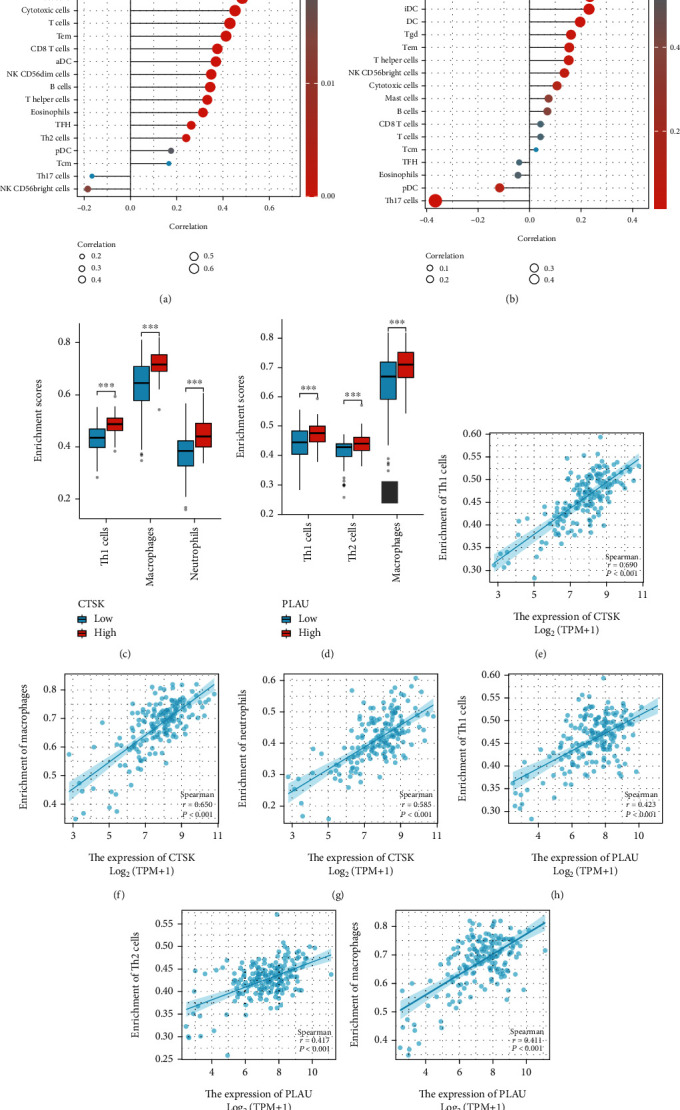
Correlation of PLAU and CTSK expression with immune infiltration level in PAAD. (a, b) Correlation between PLAU and CTSK expression and relative abundance of 24 types of immune cell. The size of the dot corresponds to the absolute Spearman correlation coefficient values. (c, d) Comparison of top 3 immune infiltration levels of immune cells between the high- and low-CTSK and PLAU expression groups. (e–j) Top 3 score cells in CTSK and PLAU.

CTSK expression was significantly positively linked with Th1 immune cell, macrophage, and neutrophil infiltration levels (Figures 9(a) and 9(c)). Moreover, the enrichment scores of Th1 cells, macrophages, and neutrophils in the CTSK high expression group were higher than those in the CTSK low expression group (all *p* < 0.001) (Figures 9(e)–9(g)). While for PLAU, Th1 cells, Th2 cells, and macrophage immune cell infiltration were positively correlated with the levels of PLAU expression (Figures 9(b) and 9(d)). Moreover, the enrichment scores of Th1 cells, Th2 cells, and macrophages in the PLAU high expression group were markedly higher than those in the PLAU low expression group (all *p* < 0.001) (Figures 9(h)–9(j)).

### 3.13. Relative Expression of PLAU in Pancreatic Cancer Cell Lines

**Figure 10:**
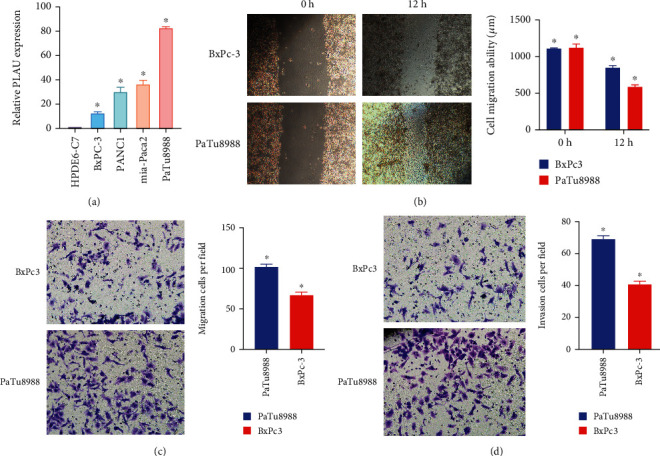
Relative expression levels of PLAU in cell lines and its effect on migration and invasion of pancreatic cancer cell lines. (a) The relative PLAU expression in PAAD cell line. (b, c) Its effect on the migration of PAAD cell lines. (d) Its effect on the invasion of PAAD cell lines.

To further identify the expression of PLAU between pancreatic cell lines and normal pancreatic cell lines, we examined the expression level of PLAU in HPDE6-C7, PaTu8988, PANC-1, BxPc-3, and mia-Paca2 cells using real-time PCR. The expression of PaTu8988, PANC-1, and BxPc-3 was significantly higher than that of human normal pancreatic ductal epithelial cell HPDE6-C7. In different pancreatic cancer cells, the expression level was also different. The expression level of the BxPc-3 cell line was relatively low, the PaTu8988 cell line was relatively high, and PANC-1 and mia-Paca2 were between the two, and the difference was statistically significant (^∗^*p* < 0.05) (Figure 10(a)).

### 3.14. PLAU Improve the Migration and Invasion of Pancreatic Cancer Cells

Scratch experiments showed that PaTu8988 cell lines had stronger migration ability than BxPc3 cell lines (Figure 10(b)). Transwell assay showed that the migration and invasion abilities of PaTu8988 were stronger than those of BxPc3, suggesting that PLAU plays a great role in the migration and invasion of pancreatic cancer (Figures 10(c) and 10(d)).

## 4. Discussion

Pancreatic cancer global incidence has significantly contributed to cancer-related deaths due to challenges in early detection and limited treatment options for advanced-stage cancers. Although carbohydrate antigen 19-9 has been FDA-approved as a serum biomarker, its limited specificity precludes its use for early detection of pancreatic cancer [16]. Thus, there is an urgent need for reliable biomarkers to facilitate the early detection of pancreatic cancer. TCGA [17] and GEO [18] databases contain vast amounts of clinical data that can be used for further data analysis. In this study, we analyzed three microarray databases to identify differentially expressed genes between pancreatic cancer tissues and noncancerous tissues. The results showed that COL1A1, PLAU, and CTSK were highly expressed in the three datasets, with COL1A1 having been shown to play a significant role in pancreatic cancer [19]. Therefore, we selected PLAU and CTSK as target genes for further research.

PLAU and CTSK have been shown to play a role in various tumors, with PLAU being considered as a potential biomarker in head and neck squamous cell carcinoma [10], promoting the EMT process [11], and tumor development in gastric cancer by cooperating with FOXM1 [12]. In pancreatic cancer, existing studies have shown that PLAU may be related to lymphatic metastasis [13], but its specific molecular mechanism is yet to be elucidated. While in pulmonary lymphangioleiomyomatosis, CTSK is a better prognostic marker [14], in colorectal cancer, it acts as a mediator between gut microbiota imbalance and tumor metastasis [20], and in prostate cancer, it affects tumor migration and invasion through the IL-17/CTSK axis, making it a potential therapeutic target [15]. However, the molecular mechanism and clinical prediction of CTSK in pancreatic cancer have not yet been studied.

Therefore, we used TCGA database to confirm the clinical value of these two genes including the KM curve, ROC curve, and Cox regression analysis. And our parts of methods mainly refer to similar literature on colon cancer [21]. As the molecular mechanism of CTSK and PLAU in the development of pancreatic cancer has not been studied, we identified the DEGs of these two genes in pancreatic cancer through the DsEq2 package and conducted functional studies of KEGG, GO, and GSEA enrichment. The results suggest that CTSK and PLAU are involved in the related molecular mechanism of immune infiltration.

Methylation is involved in a variety of cellular processes and human diseases, and methylation-related genes have been confirmed as potential biomarkers for early diagnosis of colon cancer [22]. M1a methylation modification has been confirmed to play an important role in the prognosis and shaping of the immune microenvironment of ovarian cancer [23]. Therefore, we performed methylation analysis of these two genes simultaneously to further validate their clinical value and predict their potential molecular mechanisms in pancreatic cancer.

Tumor microenvironments are characterized by immune cells, stromal cells, blood vessels, and the extracellular matrix, with tumors infiltrated by a variety of adaptive and innate immune cells that exert both protumor and antitumor functions [24]. Molecules that constitute the immune microenvironment, such as pyroptosis-related lncRNAs, can predict patient prognosis and immunotherapy effects [25]. Immune infiltration provides new ideas for tumor therapy [3, 26], as confirmed in malignant tumors such as breast cancer [27]. Immunotherapy, as a new tumor treatment method, is based on the principle of providing passive or active immunity for malignant tumors by using the immune system to target them, which is closely related to immune infiltration and has been widely used clinically [19], but it also faces problems such as drug resistance, similar to traditional chemotherapy. Differentially expressed genes change the composition of the microenvironment by recruiting different types of immune cells, thereby affecting the efficacy of immunotherapy. In epithelial ovarian cancer, immune-related genes screened using the CIBERSORT algorithm proved useful for the development of novel immune biomarkers and targeted therapy for epithelial ovarian cancer [23]. In pancreatic cancer, where immunotherapy has implications in it. Further studies are needed to validate the clinical significance of these biomarkers in larger cohorts of patients and to elucidate their molecular mechanisms in immune infiltration and other aspects of pancreatic cancer.

In conclusion, our study identified PLAU and CTSK as potential biomarkers for pancreatic cancer through bioinformatics analysis of microarray data. We validated their clinical significance through methylation analysis, immunohistochemistry, and survival analysis using TCGA data. Furthermore, we provided insights into the potential molecular mechanisms of these biomarkers in immune infiltration and other aspects of pancreatic cancer. Our findings contribute to the development of new therapeutic strategies for pancreatic cancer and lay the foundation for further studies to elucidate the role of PLAU and CTSK in this disease.

## Figures and Tables

**Table 1:** Function enrichment.

Ontology	ID	Description	Gene ratio	Bg ratio	*p* value	*p* adjust	*q* value
BP	GO:0030198	Extracellular matrix organisation	5/9	368/18670	3.42*e* − 07	1.37*e* − 04	7.23*e* − 05
BP	GO:0043062	Extracellular structure organisation	5/9	422/18670	6.74*e* − 07	1.37*e* − 04	7.23*e* − 05
BP	GO:0035987	Endodermal cell differentiation	3/9	45/18670	1.09*e* − 06	1.48*e* − 04	7.79*e* − 05
BP	GO:0001706	Endoderm formation	3/9	50/18670	1.50*e* − 06	1.53*e* − 04	8.06*e* − 05
BP	GO:0007492	Endoderm development	3/9	76/18670	5.35*e* − 06	4.37*e* − 04	2.30*e* − 04
CC	GO:0043256	Laminin complex	2/9	10/19717	8.32*e* − 06	1.87*e* − 04	9.38*e* − 05
CC	GO:0005604	Basement membrane	3/9	95/19717	8.91*e* − 06	1.87*e* − 04	9.38*e* − 05
CC	GO:0062023	Collagen-containing extracellular matrix	4/9	406/19717	2.06*e* − 05	2.88*e* − 04	1.44*e* − 04
CC	GO:0044420	Extracellular matrix component	2/9	51/19717	2.33*e* − 04	0.002	0.001
CC	GO:0005882	Intermediate filament	2/9	214/19717	0.004	0.028	0.014
MF	GO:0005518	Collagen binding	3/9	67/17697	4.29*e* − 06	1.41*e* − 04	6.77*e* − 05
MF	GO:0005201	Extracellular matrix structural constituent	3/9	163/17697	6.19*e* − 05	0.001	4.88*e* − 04
MF	GO:0004222	Metalloendopeptidase activity	2/9	103/17697	0.001	0.013	0.006
MF	GO:0008201	Heparin binding	2/9	169/17697	0.003	0.024	0.011
MF	GO:0008237	Metallopeptidase activity	2/9	181/17697	0.004	0.024	0.011
KEGG	hsa04510	Focal adhesion	4/6	201/8076	5.37*e* − 06	2.10*e* − 04	1.41*e* − 04
KEGG	hsa04512	ECM-receptor interaction	3/6	88/8076	2.44*e* − 05	3.63*e* − 04	2.45*e* − 04
KEGG	hsa05222	Small cell lung cancer	3/6	92/8076	2.79*e* − 05	3.63*e* − 04	2.45*e* − 04
KEGG	hsa05146	Amoebiasis	3/6	102/8076	3.81*e* − 05	3.71*e* − 04	2.50*e* − 04
KEGG	hsa04151	PI3K-Akt signaling pathway	4/6	354/8076	5.08*e* − 05	3.96*e* − 04	2.67*e* − 04

**Table 2:** Function of 12 hub genes.

No.	Gene symbol	Full name	Function
1	COL11A1	Collagen type XI alpha-1 chain	This gene encodes one of the two alpha chains of type XI collagen, a minor fibrillar collagen. Type XI collagen is a heterotrimer, but the third alpha chain is a posttranslationally modified alpha-1 type II chain. Mutations in this gene are associated with type II Stickler syndrome and with Marshall syndrome. A single-nucleotide polymorphism in this gene is also associated with susceptibility to lumbar disc herniation. Multiple transcript variants have been identified for this gene

2	ASPM	Abnormal spindle-like microcephaly-associated protein	Involved in mitotic spindle regulation and coordination of mitotic processes. The function of regulating microtubule dynamics at spindle poles including spindle orientation, astral microtubule density, and poleward microtubule flux seems to depend on the association with the katanin complex formed by KATNA1 and KATNB1. Enhances the microtubule lattice severing activity of KATNA1 by recruiting the katanin complex to microtubules. Can block microtubule minus-end growth, and reversely, this function can be enhanced by the katanin complex

3	CTSK	Cathepsin K	Thiol protease is involved in osteoclastic bone resorption and may participate partially in the disorder of bone remodeling. Displays potent endoprotease activity against fibrinogen at acid pH. May play an important role in extracellular matrix degradation. Involved in the release of thyroid hormone thyroxine (T4) by limited proteolysis of TG/thyroglobulin in the thyroid follicle lumen

4	OAS1	2′-5′-Oligoadenylate synthase 1	Interferon-induced, dsRNA-activated antiviral enzyme which plays a critical role in cellular innate antiviral response

5	PLAU	Urokinase-type plasminogen activator	Specifically cleaves the zymogen plasminogen to form the active enzyme plasmin

6	EPHA4	Ephrin type-A receptor 4	Receptor tyrosine kinase, which binds membrane-bound ephrin family ligands residing on adjacent cells, leads to contact-dependent bidirectional signaling into neighboring cells. The signaling pathway downstream of the receptor is referred to as forward signaling, while the signaling pathway downstream of the ephrin ligand is referred to as reverse signaling. Highly promiscuous, it has the unique property among Eph receptors to bind and to be physiologically activated by both GPI-anchored ephrin-A and transmembrane ephrin-B ligands including EFNA1 and EFNB3. Upon activation by ephrin ligands, it modulates cell morphology and integrin-dependent cell adhesion through regulation of the Rac, Rap, and Rho GTPases activity. Plays an important role in the development of the nervous system by controlling different steps of axonal guidance including the establishment of the corticospinal projections. May also control the segregation of motor and sensory axons during neuromuscular circuit development. In addition to its role in axonal guidance, it also plays a role in synaptic plasticity. Activated by EFNA1, it phosphorylates CDK5 at “Tyr-15” which in turn phosphorylates NGEF, regulating RHOA and dendritic spine morphogenesis. The nervous system also plays a role in repair after injury, preventing axonal regeneration, and in angiogenesis, which plays a role in central nervous system vascular formation. Additionally, its promiscuity makes it available to participate in a variety of cell-cell signaling, regulating, for instance, the development of the thymic epithelium. During development, the cochlear organ of Corti regulates pillar cell separation by forming a ternary complex with ADAM10 and CADH1 which facilitates the cleavage of CADH1 by ADAM10 and disruption of adherens junctions

7	ITGA3	Integrin alpha-3	Integrin alpha-3/beta-1 is a receptor for fibronectin, laminin, collagen, epiligrin, thrombospondin, and CSPG4. Integrin alpha-3/beta-1 provides a docking site for FAP (seprase) at invadopodia plasma membranes in a collagen-dependent manner and hence may participate in the adhesion, formation of invadopodia, and matrix degradation processes, promoting cell invasion. Alpha-3/beta-1 may mediate with LGALS3 the stimulation by CSPG4 of endothelial cell migration

8	RUNX2	Runt-related transcription factor 2	Transcription factor involved in osteoblastic differentiation and skeletal morphogenesis

9	CXCL5	C-X-C motif chemokine 5	Involved in neutrophil activation. In vitro, ENA-78(8-78) and ENA-nnn(9-78) show a threefold higher chemotactic activity for neutrophil granulocytes

10	FAM150B	Family with sequence similarity 150 member B	Enables receptor signaling protein tyrosine kinase activator activity and receptor tyrosine kinase binding activity. Involved in positive regulation of the ERK1 and ERK2 cascades, positive regulation of the ERK5 cascade, and positive regulation of neuron projection development. Predicted to be located in an extracellular region

11	CPA2	Carboxypeptidase A2	Similar to that of carboxypeptidase A (EC 3.4.17.1), but with a preference for bulkier C-terminal residues

12	GABRP	Gamma-aminobutyric acid receptor subunit pi	GABA, the major inhibitory neurotransmitter in the vertebrate brain, mediates neuronal inhibition by binding to the GABA/benzodiazepine receptor and opening an integral chloride channel. In the uterus, the function of the receptor appears to be related to tissue contractility. The binding of this pI subunit with other GABA(A) receptor subunits alters the sensitivity of recombinant receptors to modulatory agents such as pregnanolone

## Data Availability

The datasets generated and/or analyzed during the current study are available in TCGA database (https://www.cancer.gov/ccg/research/genome-sequencing/tcga) and the GEO database (https://www.ncbi.nlm.nih.gov/geo/).
